# UniBic: Sequential row-based biclustering algorithm for analysis of gene expression data

**DOI:** 10.1038/srep23466

**Published:** 2016-03-22

**Authors:** Zhenjia Wang, Guojun Li, Robert W. Robinson, Xiuzhen Huang

**Affiliations:** 1 School of Mathematics, Shandong University, Jinan, Shandong 250100, P.R. China; 2 Department of Computer Science, University of Georgia, Athens, GA 30602, USA; 3 Department of Computer Science, Arkansas State University, Jonesboro, AR72467.

## Abstract

Biclustering algorithms, which aim to provide an effective and efficient way to analyze gene expression data by finding a group of genes with trend-preserving expression patterns under certain conditions, have been widely developed since Morgan *et al.* pioneered a work about partitioning a data matrix into submatrices with approximately constant values. However, the identification of general trend-preserving biclusters which are the most meaningful substructures hidden in gene expression data remains a highly challenging problem. We found an elementary method by which biologically meaningful trend-preserving biclusters can be readily identified from noisy and complex large data. The basic idea is to apply the longest common subsequence (LCS) framework to selected pairs of rows in an index matrix derived from an input data matrix to locate a seed for each bicluster to be identified. We tested it on synthetic and real datasets and compared its performance with currently competitive biclustering tools. We found that the new algorithm, named UniBic, outperformed all previous biclustering algorithms in terms of commonly used evaluation scenarios except for BicSPAM on narrow biclusters. The latter was somewhat better at finding narrow biclusters, the task for which it was specifically designed.

Gene expression microarray data measures expression levels of transcribed mRNA and is arranged in a matrix in which genes correspond to rows and experimental conditions (samples) to columns. Each entry (a real number) represents the expression level of a gene under a specific condition. The need to analyze vast amounts of biological data, including gene expression data, has been driving the development of new data mining (especially biclustering) methods. At first, algorithms such as hierarchical clustering^1^ and k-means^2^ were investigated to identify sets of functionally related genes or conditions. These traditional clustering methods usually group genes which exhibit similar expression levels across all conditions by maximizing across-cluster variations or minimizing within-cluster variations. But genes may not co-express under all conditions. For instance, a cellular process may only affect a small set of genes under certain conditions, so that a subset of genes may be co-regulated or co-expressed under only a subset of experimental conditions. Biologically, genes which are co-regulated under a subset of experimental conditions exhibit expression patterns which are *trend-preserving*, but which may be quite different in values under those conditions. Here a gene expression pattern refers to the vector of expression values of the gene under the specific conditions. Two gene expression patterns are said to be trend-preserving if and only if their corresponding vectors are either order-preserving or order-reversing. Two vectors *x* and *y* are said to be order-preserving if and only if any two corresponding components have the same rank (with respect to the numerical value) in their respective vectors, and order-reversing if and only if *x* and -*y* (or equivalently -*x* and *y*) are order-preserving. For general purpose applicability, the entries in a row within a trend-preserving bicluster are allowed to be same. Consider the following example.

**Example 1**: A trend-preserving bicluster of three genes under seven conditions. The first and second rows are order-preserving, and the other two possibilities (first and third rows, second and third rows) are both order-reversing.
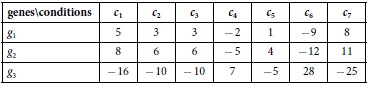


We call a bicluster order-preserving if every pair of rows is order-preserving. Obviously, any trend-preserving bicluster is either order-preserving, or else the disjoint union of two order-preserving biclusters. In example 1 these are {*g*_1_, *g*_2_} and {*g*_3_}.

Finding a maximum subset of genes of trend-preserving expression patterns under a maximum subset of conditions is impossible using traditional clustering methods. Moreover, a single gene may participate in multiple pathways under different subsets of conditions, resulting in one function pattern under one subset of conditions and a different one under another, making the problem even more challenging. Biclustering methods have been proposed with the aim of overcoming these limitations in order to uncover the genetic relationships that are not apparent. Biclustering algorithms have been widely developed since Morgan *et al.*^3^ pioneered a work on partitioning a matrix into submatrices with approximately constant values. Cheng and Church^4^ were the first to apply the biclustering idea to analyze gene expression data. Since then research on biclustering algorithm development in bioinformatics has focused on this application. Existing biclustering algorithms can be grouped into five categories in terms of the techniques on which they are based^5^:

1. Iterative row and column clustering combination: row clusters are combined with column clusters and vice versa, e.g. Interrelated Two-Way Clustering^6^ and Coupled Two-Way Clustering^7^;
2. Divide and conquer: the problem is recursively broken down into checkerboard sub-problems, e.g. BiMax^8^, Hartigan^9^;
3. Greedy iterative search: locally optimal results are chosen in hopes that they might be globally optimal, e.g. Cheng and Church^4^, the Flexible Overlapped biclustering algorithm^10^, xMOTIFs^11^;
4. Exhaustive bicluster enumeration: enumerating all the possible biclusters, e.g. SAMBA^12^, OP-Cluster^13^;
5. Distribution parameter identification: biclusters are assumed to follow a given statistical model and parameters are identified to fit in the best way, e.g. Spectral biclustering methods^14^, Plaid^15^ and Sheng *et al.*^16^.

Each of these biclustering algorithms is restricted to specific types of biclusters and datasets. In the assessment of twelve biclustering algorithms on twenty synthetic datasets from six models^17^, each algorithm performed well on one or a few datasets, but none performed well on all of them. With the availability of more and more microarray datasets, it has become important to develop a comprehensive biclustering algorithm to analyze gene expression data. In this article we present an elementary method for biclustering. Our method substantially overcomes the limitations of all prior biclustering algorithms, and enables discovery of the most biologically meaningful biclusters in gene expression datasets.

Biologically speaking, trend-preserving biclusters are the most meaningful local structures hidden in a data matrix. Trend-preserving biclusters are a generalization of all widely studied types of biclusters, including constant, shift, scale, and shift-scale biclusters. The latter two types of biclusters were ever considered computationally challenging to identify^18^.

Ben-Dor *et al.*^19^ developed an algorithm (OPSM) to discover significant order-preserving biclusters based on statistical strategies. In their model, the rows of the input matrix are required to be permutations of some *m* positive integers, *1*, *2*, …, *m*, as well as to be pairwise different. The technique used in OPSM is essentially an exhaustive approach by iteratively growing each possible submatrix based on statistical evaluations. It has proved unsatisfactory to apply OPSM to the analysis of gene expression datasets^17^. Ever since many methods have been proposed to mine frequent sequential patterns as the extension of the OPSM approach, e.g. OPSM-RM^20^ collects results from repeated experiments to cope with noise, GeBOPSM^21^ proposes a generalized OPSM model by relaxing the requirement that each row has to be composed of different integers in OPSM, and POPSM^22^ captures similar local correlations in probabilistic matrices with uncertain data. However in these models the optimal solutions may not be guaranteed as long patterns with few supports might be pruned in early stage and the requirements of computational resource are explosive. Jiang *et al.*^23^ also proposed a parallel partitioning and mining method based on Butterfly Network to extend and improve OPSM.

The biclustering algorithm QUBIC^24^ we previously developed attempts to discover trend-preserving biclusters in gene expression data by granulating gene expression values into *r* ≥ 1 ranks. However, its performance degrades rapidly as the number of ranks of gene expression values increases. Example 2 shows what is wrong with QUBIC for an example of a bad case.

**Example 2**: bad case for QUBIC


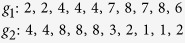


Obviously, expression patterns (2, 2, 4, 4, 4) of *g*_1_ and (4, 4, 8, 8, 8) of *g*_2_ under conditions 1, 2, 3, 4, 5 are order-preserving. After ranking as in QUBIC (for any *r* > 0), these two order preserved patterns could no longer be identified, which leads to an incorrect result. If *r* = 1 then all ranks are 1, so the whole 2 × 10 array is outputted as a bicluster, but it is not meaningful. If *r* = 2, the pattern becomes


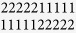


which contains only the empty bicluster. Similarly, QUBIC recognizes only the empty bicluster in (*g*_1_, *g*_2_) for any *r* > 1. Our observation, which is very natural, leads to a Universal approach for discovering trend-preserving Biclusters in gene expression data, which is based on an application of the longest common subsequence (LCS) algorithm^25^ to a new matrix derived from the input data matrix. We tested and compared UniBic with six other competitive biclustering algorithms, including OPSM^19^, QUBIC^24^, ISA^26^, FABIA^27^, CPB^28^, and BicSPAM^29^, on large scale synthetic and real datasets. The comparison results demonstrate that UniBic overwhelmingly outperforms all of them with an exception that it is inferior to BicSPAM only when finding extremely narrow biclusters because BicSPAM was specifically designed for this purpose.

## Results

### Algorithm Validation

To evaluate the biclustering algorithm UniBic, we compared it with six currently popular biclustering algorithms, including OPSM^19^, BicSPAM^29^, QUBIC^24^, ISA^26^, FABIA^27^ and CPB^28^, on both synthetic and real datasets. Biclustering algorithms developed based on different methods tend to perform differently on various datasets, while some algorithms may perform better on one kind of datasets, others may tend to be better on other kinds of datasets. In order to fairly evaluate these algorithms, we tested them on six different types of synthetic datasets and eight real datasets from GEO database^30^ with the aid of BiBench framework^17^.

### Validation on synthetic data

As the biclusters to be discovered in synthetic data are supposed to be known, we compared identified biclusters with the genuine ones. Let *b*_1_ and *b*_2_ be two biclusters, their similarity is measured by Jaccard coefficient^31^:


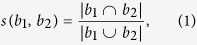


where |*b*_1_ ∩ *b*_2_| is the number of genes in their intersection, and |*b*_1_ ∪ *b*_2_| is the number of their union. For two sets of biclusters *M*_1_ and *M*_2_, the similarity score between them is calculated using the formula introduced in^8^:


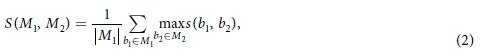


which measures the average similarity of biclusters in *M*_1_ with the biclusters in *M*_2_. *Recovery score* is defined as *S*(*G*, *D*), and *relevance score* as *S*(*D*, *G*), where *G* and *D* represent the sets of genuine biclusters and discovered biclusters, respectively.

### Validation on GDS data

Evaluation of results on real data is different from on synthetic data as the genuine biclusters are not known. We validated biclusters by calculating the Gene Ontology enrichment (a statistically significant test which describes the probability of a gene set containing a certain number of particular GO terms) for genes in the discovered biclusters. This functional analysis was carried out using the GO stats package^32^. A bicluster here is assumed to be enriched if it has at least one GO term with statistically significant *p*-value smaller than 0.05, where the *p*-value is adjusted by multiple test correction using the method from Benjamini and Hochberg^33^ (a way to control the false discovery rate in large datasets to reduce the number of false positives).

### Testing on Synthetic Datasets

We first tested UniBic and the other six tools on synthetic datasets. All the algorithms were executed with their optimized parameters, respectively. For the test on synthetic datasets, there was no need to run the preprocessing steps (see Methods for more details) since all of the synthetic data was to be treated as genuine. To skip over the preprocessing steps, UniBic simply ran with parameters *q* = 0.5 and *r* = *m*. Here *m* is the number of columns in the input data matrix.

Six different types of synthetic datasets were generated. The background matrices of synthetic datasets are of given large size with entries randomly chosen from Gaussian distribution *N*(0, 1), then given smaller sized submatrices were chosen with entries modified in according to the rules presented for Types I–VI, which are type I: trend-preserving biclusters; type II: column-constant biclusters; type III: row-constant biclusters; type IV: shift-scale biclusters; type V: shift biclusters; type VI: scale biclusters. Type I is biologically the most meaningful type and is a generalization of the others. It is generated by randomly selecting a base row within a selected submatrix and then rearranging the entries in other rows of the submatrix so that the rearranged submatrix is trend-preserving. Type II is generated by randomly selecting a row within a selected submatrix, and copying it to other rows in this submatrix. Type III is generated by randomly selecting a column within a selected submatrix, and copying it to other columns in this submatrix. Type IV is generated by randomly selecting a row within a selected submatrix as a base row, and replacing the other rows of the submatrix by both shifting and scaling of the base row. Type V is generated as in the type IV but with scaling parameter 1. Type VI is generated as in the type IV but with shifting parameter 0.

### Comparison on six types of biclusters

**Figure 1:**
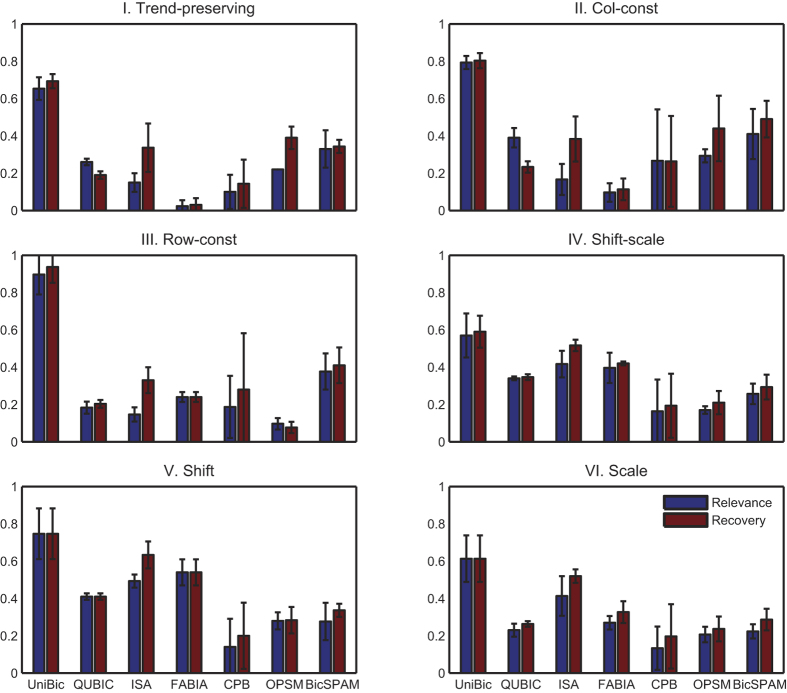
Relevance and recovery scores of the seven algorithms on six types of biclusters, with error bars.

To begin with, we generated at random three kinds of non-overlapping test matrices: a) matrices of size 150 × 100 with three implanted biclusters of size 15 × 15; b) matrices of size 200 × 150 with four implanted biclusters of size 20 × 20; c) matrices of size 300 × 200 with five implanted biclusters of size 25 × 25. Five rows were selected as order-reversing rows versus the base row for type I biclusters, and for all the six types of biclusters, five datasets were generated for each kind of test matrix through repetition. The average relevance and recovery scores among all test matrices are shown in Fig. 1 for each tool.

On type I test matrices with trend-preserving biclusters implanted, UniBic overwhelmingly outperformed all other competitive algorithms with an average relevance score of 0.65 compared to the second highest relevance score of 0.33 for BicSPAM, and with an average recovery score of 0.69 versus the second highest recovery score of 0.39 for OPSM. The results show that UniBic discovers trend-preserving biclusters in data array much better than any of the other six tools. The data shows even greater advantages for UniBic with type II and type III test matrices. On the other test matrices, the advantage of UniBic was comparatively less significant, but it still outperformed all the other algorithms. Taken together, the comparisons indicate that UniBic is more stable than any of the other six algorithms, as well as performing better.

OPSM performed best on type I and type II test matrices, in which the values are strict order-preserving with relatively large value gaps between bigger and smaller values in each row of implanted biclusters. On the type III test matrices, where the entries in each row of implanted biclusters are consist with a constant value, its performance became rather poor. BicSPAM performed slightly better than OPSM on almost all types of test matrices, and as it allows equal values in matrices, it performed well on type III test matrices. QUBIC performed well on type IV and V test matrices with large *q* values because it is suitable for datasets with biclusters which can be granulated to be separated from the background data. ISA and FABIA both showed their best performances on type V test matrices, as they were designed to perform well on datasets generated from data distribution with large variances, and their performances on type IV and type VI test matrices were also better than on other test matrices. CPB showed the least stable performance in repetitive experiments compared with other tools as it starts with a randomly selected set of columns, leading to significant fluctuations in its output.

The comparison results shown in Fig.1 demonstrate that UniBic does overwhelmingly outperform all the compared tools on the datasets with implanted biclusters of nearly square shape. Our original intention in development of biclustering algorithms is to seek those biclusters of (nearly) square shape just like most computational scientists did. However, the narrow biclusters, with huge number of rows but only a few columns, are usually more important to biologists. Simultaneously with the development of the UniBic, we noticed BicSPAM^29^, which was designed specifically for extremely narrow biclusters, e.g. of rows more than 200 and columns less than 8. In this extremely situation, BicSPAM performs somewhat better than UniBic as it was mentioned in BicSPAM^29^ that biclustering algorithms which are designed on the adoption of maximal sequential patterns may to some extent overlook narrow biclusters.

To evaluate the capability of finding narrow biclusters of UniBic, we further tested it on the datasets with narrow biclusters implanted compared with six other algorithms. Comparison results from Supplementary Fig. S1 show that UniBic overwhelmingly outperforms all the competitive ones, including BicSPAM, unless the to-be-identified biclusters are very narrow, and its performance is almost independent of the number of rows. When the implanted biclusters become very narrow, e.g. with less than 8 columns but with more than hundreds of rows, the algorithm BicSPAM is more capable of returning accurate results as it is specially designed to identify this kind of narrow biclusters. However, BicSPAM’s performance rapidly degrades as the columns of the to-be-identified biclusters increase in number. It is worthy stressing that the version of BicSPAM we compared with in this article is in the absence of enhancements to foster the scalability of the underlying pattern mining searches and to deal with large scale datasets. The improved version^34^ of BicSPAM has been developed for further integration of network information into its biclustering procedure.

### Comparison on overlapping biclusters

Then we tested the seven tools on synthetic datasets with overlapping biclusters. The overlapping biclusters were generated by replacing the selected biclusters with trend-preserving biclusters. Four kinds of synthetic matrices were generated with three implanted biclusters overlapped of size 0 × 0, 3 × 3, 6 × 6 and 9 × 9 respectively, where the background matrices are of size 200 × 150 and biclusters are of size 20 × 20. Values in each of the three selected biclusters were shifted with 2, 4 and 6 to ensure that they were still trend-preserved while overlapped. Repeating the procedure five times, we obtained five synthetic matrices for each overlapping size.

**Figure 2:**
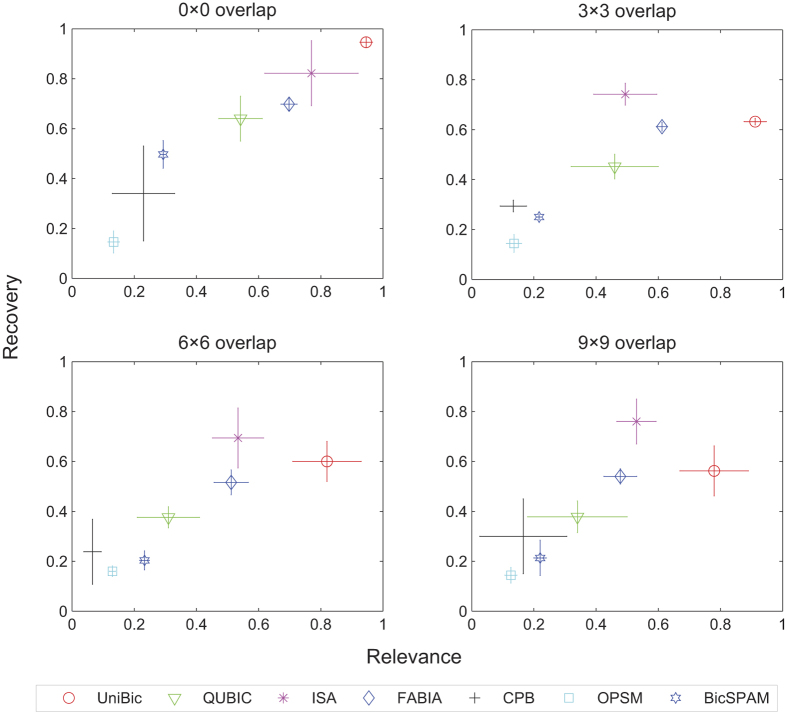
Relevance and recovery scores of the seven algorithms on synthetic matrices with overlapping biclusters, including error bars.

The relevance and recovery scores of seven algorithms on each kind of the test matrices with overlapping biclusters are shown in Fig. 2. The results showed that for most algorithms, their performances went down as the degree of overlap increased. OPSM’s scores did not change much as its initial scores were low. ISA and FABIA showed robust performances with high scores. Our UniBic found nearly all the implanted biclusters when the biclusters were not overlapped. Although our performance was affected when the biclusters were overlapped, it still found most of the implanted biclusters, and the result did not change much when the overlapping size increased. This indicates that UniBic is more capable of finding overlapping biclusters than other compared tools.

### Testing on Real Datasets

We further tested the seven tools on the eight gene expression datasets GDS181, GDS589, GDS1406, GDS1451, GDS1490, GDS2520, GDS3715, GDS3716 from the GEO database^30^. The detailed description of these datasets is summarized in Table 1.

Considering the inactive entries and noise interference, we first preprocessed all the datasets (see Methods for more details). Up- and down-regulated values were separated from the background data with parameter *q* to be 15/*m* and *r* to be 15. For other algorithms that are required to be run on array data without missing values, the PCA imputation^35^ was carried on the expression datasets, and all the algorithms were run with their parameters optimized. The GO enrichment was evaluated for each bicluster discovered by each tool, with significant *p*-value 0.05. Since different algorithms are based on different theoretic models, and their best performances with respectively optimized parameters may lead to different number of output biclusters, we assessed their performances by the proportion of GO enriched biclusters. All the statistics are shown in Table 2. UniBic outputted 62 enriched biclusters from 151 discovered ones, and reached the highest average enrichment level of 41.1% of these eight datasets. FABIA showed 22 enriched biclusters from 80 discovered ones, reached the lowest average enrichment level. ISA discovered the most biclusters of 217, but with a comparative lower average enrichment level of 32.7%. OPSM found the second most biclusters, but still with a comparative lower average enrichment level of 29.5%. QUBIC and CPB both had relatively higher average enrichment levels. We ran BicSPAM on the same real datasets, but we did not get final results because it was always out of memory.

### Utilization of Computing Resources

Biclustering has been well known to be computationally intractable, and therefore it is highly challenging to develop an effective and efficient heuristic algorithm in order to meet the needs of analyzing large data matrices. Taking the total number of CPU operations required as the measure of time, we see that UniBic takes *O*(*nm*log*m*) times to create the index matrix, *O*(*q*^2^*n*^*2*^*m*^2^*/k*) to locate seeds of to-be-identified biclusters, and *O*(*q*^2^*m*^2^*n*^2^) to extend biclusters. Thus the overall running time of UniBic is up bounded by *O*(*q*^*2*^*m*^*2*^*n*^2^), from which we see that the running time of UniBic is independent of size of the biclusters to be identified, and even almost independent of columns of input matrix because *qm* approaches a constant value.

**Figure 3:**
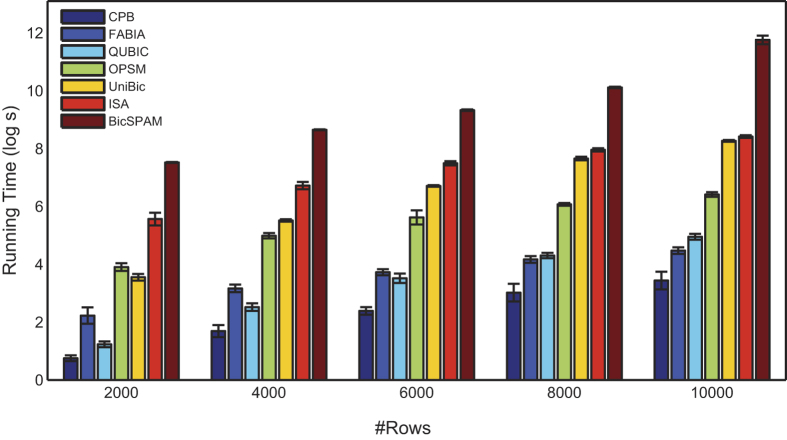
Comparison of the distributions of running time for the seven algorithms versus the number of rows on the matrices of 50 columns, with error bars. The time scale is logarithmic.

To compare the computing resource usage for different algorithms, we ran the seven tools on the test matrices with fixed number of 50 columns, and calculated the individual running time distributions of the seven algorithms with their respective default parameters versus the number of rows. The algorithms were tested on these large test sets on a desktop computer (2.66 GHz Inter Core, 2 Duo CPU, and 4 GB memory). Figure 3 displays the comparison results among the seven individual running time distributions versus the number of rows.

## Discussion

Since the first biclustering strategy was pioneered by Morgan *et al.*^3^ in 1963, researchers have attempted to develop an effective and efficient algorithm capable of discovering trend-preserving biclusters. Various biclustering algorithms have been playing important roles in the analysis of gene expression data, but the identification of general trend-preserving biclusters remains a challenging problem. Intuitively, as is also mentioned in^19^, the key to discovering biclusters in a data matrix lies in predicting a seed for each significant (trend-preserving) bicluster hidden in the data matrix to be analyzed. It has been considered to be rather challenging^19^ even in the special case where input matrix consists of *n* distinct permutations of (*1*, *2*, …, *m*). The UniBic captures the essence of how to locate a seed of each to-be-identified bicluster hidden in a background matrix by finding a longest common subsequence between two rows of the index matrix derived from the input matrix. This provides a transformation from the problem of discovering trend-preserving biclusters in a background matrix to a simple problem of finding the longest common subsequence between two rows of the index matrix derived from the background matrix. This transformation may seem to be routine, but it does improve the traditional biclustering approaches. Methodologically, UniBic takes an essential step towards the identification of the most general and meaningful biclusters hidden in a noisy and complex data matrix. The results on both synthetic and real data sets demonstrate that UniBic is more promising in discovery of functionally correlated expression patterns in gene expression data, and proves to be a powerful biclustering analysis tool for large microarray data.

## Methods

In this section, we present our novel biclustering algorithm, which is capable of discovering all the significant trend-preserving biclusters hidden in a data matrix. The basic idea behind the algorithm comes from the following observations: 1) there exists a column permutation of an order-preserving bicluster such that the entries of each permuted row within the bicluster are increasingly (not necessarily strictly) arranged, and 2) the key to biclustering is the accurate prediction of the columns of each to-be-identified bicluster. Motivated by these two observations, we designed a novel algorithm by applying the LCS algorithm to selected pairs of rows of an index matrix derived from the input data matrix.

The foundation of the algorithm is the fact that if two rows of the input matrix *A* belong to a significant order-preserving bicluster, then the corresponding two rows of the index matrix *Y* will contain a significant common subsequence with a high probability, and vice versa. This elementary observation leads to a novel method to identify a seed for each potential trend-preserving bicluster. To achieve this goal, we could calculate all the significant common subsequences by applying the LCS algorithm to each pair of rows of *Y*. Instead, we identify a number *k* (see Supplementary Section 1 online) such that every significant order-preserving bicluster *B* must contain at least *k* + 1 rows. Now assume that *B* is such a bicluster, if we equally partition the set of rows of *A* into *k* subsets of rows, then there must be at least two rows of *B* falling into one of these *k* subsets, and the two rows are sufficient to locate a seed for *B*. Therefore, applying the LCS algorithm to each pair of rows in each of the *k* subsets of *Y* would be sufficient to anchor a seed for each significant order-preserving bicluster of more than *k* rows. This process identifies a seed for each potential bicluster hidden in the data matrix. The algorithm follows the steps below in order:

### Algorithm UniBic

#### Step 1. Index matrix creation

Let *Y* = {*y*_*ij*_} be the index matrix derived from input matrix *A* = {*a*_*ij*_} by setting:


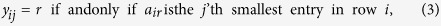


where ties are broken based on the rule that the smaller column index has higher priority to be ranked (see Supplementary Section 4 online).

#### Step 2. Index matrix partition

We calculate an integer *k* based on the significance (default set to 0.05) of the to-be-identified trend-preserving biclusters using the techniques developed in^19^. We then equally partition *Y* into *k* subsets of rows.

#### Step 3. Application of LCS

Apply the LCS algorithm to each pair of rows in each of the *k* subsets of *Y* to find all the significant longest common subsequences. For each pair of rows having a significant longest common subsequence, one such subsequence is chosen as a seed to which steps 4, 5 and 6 are to be applied. They are listed in decreasing order in length with the longest one at the front.

#### Step 4. Strict order-preserving bicluster development

We start with a longest seed at the front of the seed list obtained from step 3. The LCS algorithm is then repeatedly applied to find a 3 × *C* order-preserving submatrix of *A*, where two of the rows are from the seed and the value of C is as large as possible. We continue to add rows one at a time in a greedy fashion until the order-preserving submatrix has more rows than columns, at which point the submatrix from the previous stage is passed on to step 5.

#### Step 5. Extension to an approximately trend-preserving bicluster

From the strict order-preserving bicluster obtained in step 4, we extend it by first repeatedly adding new columns one at a time with an error rate *r* ≤ 0.3 until none is available. Up to now, the bicluster obtained is order-preserved. To identify a significant trend-preserving bicluster, we have to get those remaining original rows and their negative ones involved in the row extension process by repeatedly adding new rows (original or negative) one at a time with an error rate ≤0.15 until none is available. The row extension would be achieved by applying the LCS algorithm between the common (consensus) sequence of the column extended order-preserving bicluster and the corresponding index row in *Y* or its reverse row when we consider negative rows to be added. Then remove from the current seed list those with two corresponding rows belonging to discovered biclusters. Repeat step 4 for the next potential trend-preserving bicluster until the list is exhausted.

#### Step 6. Output as many trend-preserving biclusters as the user needs

We calculate the significance value for those trend-preserving biclusters obtained in step 5. Those with *p*-values less than 0.05 are decreasingly ordered in their significance. Then UniBic outputs first *o* trend-preserving biclusters, where *o* is a parameter which can be pre-specified by users with a default set to 100.

**Example 3:** Illustrates how to locate an initial seed of a trend-preserving bicluster in the input matrix *A*.

**Example 3**: Illustration of locating an initial seed.

**Example 3a:** Input matrix *A*:with entries of two rows and eight columns.
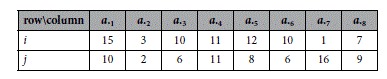


**Example 3b:** The index matrix Y of A: with entries being obtained based on eq. (3).
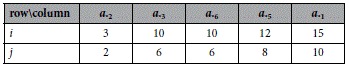


**Example 3c:** Initial seed: obtained by the longest common subsequence (2, 3, 6, 5, 1) through applying the LCS algorithm between rows *i* and *j* in *Y*.
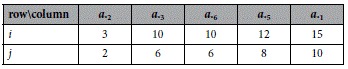


### Data Preprocessing

When the algorithm UniBic is applied to real data matrices, especially gene expression data, it is better to preprocess the input data to alleviate the adverse impacts to data entries since corresponding genes are not activated under all conditions and there is noise interference from data approximation.

### Data separation

The values of interest are usually hidden in a massive data matrix to be analyzed. Of interest in gene expression microarray matrix are those entries representing genes up- or down-regulated under corresponding conditions, which are usually only a small portion of the whole data matrix. Biologically, up- (down-) regulated expression values tend to be comparatively bigger (smaller). Those middle values which represent genes being inactive under corresponding conditions are comparatively less important in the analysis of gene expression data. Therefore, it is helpful to distinguish the values of interest from others in gene expression microarray data matrices. To do so, we chose a percentage parameter *q* with the default value set to 15/*m* (the value *q* = 0.5 is specially provided for data without separation preprocessing), where *m* is the number of columns of the input matrix, and we selected entries with values significantly away from the median value in each row of input matrix *A* as up- (down-) regulated values as follows:

1. Entries in each row *i* of *A* are increasing ordered: *a*_*i1*_, …*a*_*is*_, …*a*_*il*_, …*a*_*it*_, …*a*_*im*_, where *s* = *qm*, *l* = *m*/2 and *t* = (1− *q*)*m*, *d* = min{*a*_*il*_− *a*_*is*_, *a*_*it*_ − *a*_*il*_}.
2. For the values bigger than *a*_*il*_ + *d*, they are treated as up-regulated values, and values smaller than *a*_*il*_–*d* are treated as down-regulated values.
3. Set all the other entries in *A* to be zero, and denote by *A*’ the resultant matrix.

### Data granulation

Data array, e.g. gene expression microarray data, generated from wet laboratory is inevitably approximated, leading the algorithms, including UniBic, to be affected adversely to some extent. To avoid suffering from this approximation, we further preprocessed the input data by equally partitioning all the up-regulated decreasingly ordered entries in each row of *A*’ into *r* (a parameter which may be pre-specified by user) intervals, then we set all the entries belonging to the *i*’th interval to be the integer *i*, while the down-regulated increasingly ordered entries in each row of *A*’ were also separated into *r* intervals and entries belonging to the *i*’th interval were set to be the integer −*i*, then we get a new integer matrix denoted by *A*”.

Obviously, the trend-preserving biclusters in *A*” equivalently correspond to those in *A*. Therefore, we may apply the UniBic on *A*” to discover all the significant trend-preserving biclusters hidden in *A*. This approach has been experimentally proved to be helpful in reducing adverse impacts on performance.

## Additional Information

**Data Availability**: The source code as well as all datasets and results are available at: http://sourceforge.net/projects/unibic/files/?source=navbar. 

**How to cite this article**: Wang, Z. *et al.* UniBic: Sequential row-based biclustering algorithm for analysis of gene expression data. *Sci. Rep.* **6**, 23466; doi: 10.1038/srep23466 (2016).

## Supplementary Material

Supplementary Information

## Figures and Tables

**Table 1:** Description of GDS datasets.

Dataset	Genes	Samples	Description
GDS181	12626	84	Large-scale analysis of the human Transcriptome
GDS589	8799	122	Multiple normal tissue gene expression across strains
GDS1406	12488	87	Brain regions of various inbred strains
GDS1451	8799	94	Toxicants effect on liver: pooled and individual sample comparison
GDS1490	12488	150	Neural tissue profiling
GDS2520	12625	44	Head and neck squamous cell carcinoma
GDS3715	12626	110	Insulin effect on skeletal muscle
GDS3716	22283	42	Breast cancer: histologically normal breast epithelium

**Table 2:** The results of GO enrichment analysis on eight GDS datasets.

Algorithm	Found	Enriched
UniBic	151	62(41.1%)
OPSM	163	48(29.5%)
QUBIC	91	34(37.4%)
ISA	217	71(32.7%)
FABIA	80	22(27.5%)
CPB	96	34(35.4%)
