# Supplementary material for: UniBic: Sequential row-based biclustering algorithm for analysis of gene expression data
Source: Sci Rep. 2016 Mar 22;6:23466. doi: 10.1038/srep23466 (PMC4802312; doi:10.1038/srep23466)
Supplement: Supplementary Information [file srep23466-s1.pdf]

Supplementary Material for

UniBic: Sequential row-based biclustering algorithm for analysis of gene expression data

Zhenjia Wang[1, †], Guojun Li[1, †,*], Robert W. Robinson[2], Xiuzhen Huang[3, *]

[1]School of Mathematics, Shandong University, Jinan, Shandong 250100, P.R. China

[2]Department of Computer Science, University of Georgia, Athens, GA 30602, USA

[3]Department of Computer Science, Arkansas State University, Jonesboro, AR72467

Section 1: Statistically significant *k* value of biclusters

For an input matrix *A* of size $n \times m$, the probability that *A* has a trend-preserving sub-matrix of size $k \times s$ is estimated by the technique established in [1]. For a randomly selected row, the probability that the entries in its *s* columns support a given column index permutation is (1/*s*!). As each row is independent with each other, and there are $m \cdots (m\text{-}s+1)$ ways to choose a complete model of size *s*, the probability of having *t*=*k*+1 rows supporting the column index permutation is estimated as:

$$U = m \cdots (m - s + 1)\binom{n}{t}\left(\frac{1}{s!}\right)^{t}\left(1 - \frac{1}{s!}\right)^{(n-t)}$$

Supplementary Table S1. The row threshold of *t* for different column value *s*, when *U*<0.05.

size \ *s*	*s*=6	*s*=7	*s*=8	*s*=9	*s*=10	*s*=11	*s*=12	*s*=13	*s*=14	*s*=15	*s*=16
n=1000, *m*=50	16	10	7	6	5	4	4	4	3	3	3
n=5000, *m*=50	32	16	10	7	6	5	4	4	4	3	3
n=10000, *m*=50	47	20	12	8	6	5	5	4	4	4	3
n=15000, *m*=50	59	24	13	9	7	6	5	4	4	4	3
n=1000, *m*=100	18	11	8	7	6	5	5	4	4	4	4
n=5000, *m*=100	35	18	11	8	7	6	5	5	4	4	4
n=10000, *m*= 100	50	22	13	9	7	6	5	5	4	4	4
n=15000, *m*=100	63	26	15	10	8	6	6	5	5	4	4

n=1000, *m*=150	19	12	9	7	6	5	5	5	4	4	4
n=5000, *m*=150	36	19	12	9	7	6	6	5	5	4	4
n=10000, *m*=150	52	24	14	10	8	7	6	5	5	4	4
n=15000, *m*=150	66	27	15	11	8	7	6	5	5	5	4

From the table we can see that in real data, when the input matrices are of about 10,000 rows, *k*=3 or 4 would be sufficient for us to anchor a seed.

Section 2: Parameters for algorithms

Algorithms were run with their parameters optimized in our experiments. The parameters used for each algorithm on synthetic data are listed below in Supplementary Table S2, and on real data are listed in Supplementary Table S3. For parameters that are listed with ranges in tables, we did several experiments with different combinations to get their best performances on different datasets.

Supplementary Table 2. Parameters selected for each algorithm on synthetic data, where *m* denotes the number of columns of the input matrix.

Algorithms	Parameters
UniBic	quantile=0.5, ranks=*m*, nblocks=3-5
OPSM	lValue=3-5
BicSPAM	itermizer=4, 7, 10, 20, mincolumns=4-6
QUBIC	quantile=0.06-0.45, ranks=1~2
ISA	no_seeds=3-15
FABIA	p=3-5
CPB	n=3-5

Supplementary Table 3. Parameters selected for each algorithm on real data.

Algorithms	Parameters
UniBic	quantile=15/*m*, ranks=15
OPSM	default parameters
BicSPAM	itermizer=4, 7,10,20, mincolumns=4

QUBIC	default parameters
ISA	no_seeds=100-300
FABIA	p=10-30
CPB	n=10-30

Section 3: Results on datasets with narrow biclusters

Considering the complexity of biclustering problem, it quite difficult to design algorithms that can accurately identify biclusters of all kinds. Algorithms, include UniBic, which are designed based on the adoption of maximal row sequential are more capable of finding square shaped biclusters, but may to some extent overlook the narrow biclusters with significant number of rows but with only few columns, while algorithms including BicSPAM, which mine solutions from simple sequential patterns, are more powerful on datasets with narrow biclusters, but at the cost of losing those biclusters of fairly many columns. It's already shown in our main manuscript that UniBic overwhelmingly outperforms other algorithms on datasets with square shaped biclusters implanted. Here we also evaluate its performance of discovering narrow biclusters versus BicSPAM/OPSM since they're specially suitable for discovery of narrow ones. Supplementary Figure S1 shows the comparison results among different algorithms on synthetic datasets with trending-preserving narrow biclusters. The test matrices are of size 1000×100, with implanted biclusters of 100 rows and different columns of 10/20/30. All the results of UniBic were run with default parameters (q=0.5, r=#columns), and other algorithms were also tested with their parameters optimized with multiple repetition.

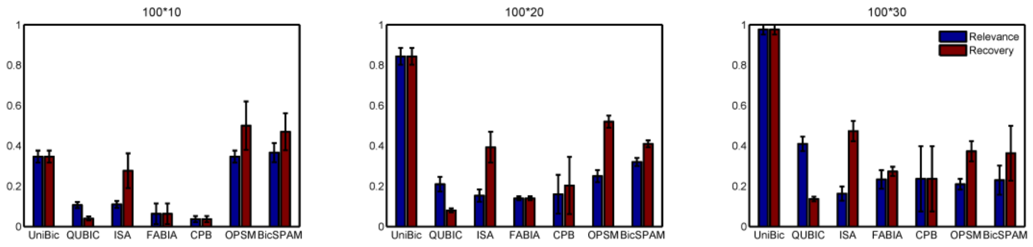

Supplementary Figure S1. Relevance and recovery scores of algorithms on synthetic datasets

with narrow biclusters, with error bars.

From the results we can see that when the biclusters are of 20 or more columns, UniBic overwhelming outperformed all other algorithms, and when the biclusters are of 30 or more columns, all the implanted biclusters could be identified by our algorithm. Although the decrease of columns negatively affected the performance of UniBic, it also got a relatively high score when the biclusters are of 10 columns. Actually when the biclusters are of less than 8 columns, it would be difficult for us to accurately capture the seeds hidden in the matrices, thus lead to worse performance compared with BicSPAM and OPSM.

BicSPAM and OPSM are more suitable for biclusters with few columns and significant number of rows, and proved to be competitive options on narrow biclusters, but their abilities to obtain accurate results in large scale biclusters are limited. The scores of BicSPAM decrease as the increase of columns in the biclusters, and it deserves to be stressed that BicSPAM will be corrupted whenever the number of columns of the to-be-identified biclusters goes beyond 30, while UniBic performs better and better as the number of columns of the to-be-identified biclusters goes up, along with a slight increase in running time.

It can be seen that other compared algorithms also perform better as the number of their columns goes up. It is worthy to stress that the trend-preserving biclusters implanted in the test matrix are almost with different values in each row of the biclusters, thus lead to a fairly good performance of OPSM compared to BicSPAM.

Section 4: Algorithm Unibic

In order to help the readers to further understand our algorithm, we describe it in more details as follows:

***Step 1. Index matrix creation*:** Create an permutation matrix $X = \{x_{ij}\}$ by rearranging the entries of each row of the input matrix $A = \{a_{ij}\}$ in increasing order with ties being broken based on the rule that smaller column index has higher priority, where $x_{ij} = a_{ii_j}$ determines the permutation of columns, and generate an index matrix $Y = \{y_{ij}\}$

with y_{ij} being the column index i_j of a_{ii_j}, i.e., $y_{ij} = i_j$;

***Step 2. Index matrix partition*:** Calculate the value *k* based on the significance of the to-be-identified trend-preserving biclusters as described in **Section 1**, and equally partition the set of rows of *Y* into *k* subsets;

***Step 3. Application of LCS*:** Apply LCS algorithm to each pair of rows of each of the *k* subsets of *Y* to find all the significant longest common sub-sequences which are listed in decreasing order in length with the longest one at the front;

***Step 4. Strict order-preserving bicluster development*:** Take a longest common subsequence on top of the current list obtained in step 3 to anchor a seed of a potential trend-preserving bicluster hided in *A*. The seed consists of two rows in the input matrix *A* from which the common subsequence originates. For a selected seed $e=g_ig_j$, an initial bicluster *B*(*I*, *J*) is built, where *I* being the subset of rows *i* and *j*, *J* being the subset of columns in their LCS. A consensus sequence *T* for current bicluster is saved and is initially identified as the LCS of g_i and g_j. The bicluster is expanded by adding genes one by one whose LCS with *T* is of most consistency. Let *T*' be the sequence in which the total consistency is maintained after adding a new gene, and let *B*'=(*I*', *J*') be the new bicluster, where *I*' is the set of *I* after adding the new row, and *J'* is the set of columns in *T*'. If *min*{|*I*'|, |*J*'|}>*min*{|*I*|, |*J*|}, set *B* to *B*'. Then we get a *block B* which is of total consistency.

***Step 5. Extension to an approximately trend-preserving bicluster*:** In less strict requirements, the block seed would be expanded by adding columns and genes without the consistency level fall below *c* as follows (*c* is a parameter provided for the users to control the tolerance of trend preserving in the bicluster expanding process with default value of 0.85): for genes listed as g_1, g_2, …, g_t in the block B, we record the *i*-1 sets of columns of the LCS of g_s (s=2,3....*t*) and g_1, and for each column that is not in *T* but appears more than certain times (for example 0.7*(|*I*|-1)) in the *i*-1 sets, we add them back into *T*, and expand the bicluster by adding those columns in the matrix. Then the current bicluster is further expanded by adding genes whose LCS with *T* is of length more than *c*|*T*|. For the genes that are negatively regulated with the seed genes, they could be extended by considering their corresponding rows reversed. Then remove from the current list those each of which has its two

corresponding rows belonging to discovered biclusters, and repeat step 4 for the next potential trend-preserving bicluster until the list is exhausted.;

***Step 6. Output as many trend-preserving biclusters as user want*:** Output the current biclusters obtained in step 5 in terms of whether or not they are statistically significant (with default value 0.05).

References:

1 **Ben-Dor, A., Chor, B., Karp, R. & Yakhini, Z. Discovering local structure in gene expression data: the order-preserving submatrix problem. Journal of computational biology 10, 373-384 (2003).**
